# Belonging in Hospital Medicine: Insights From Mapping Hospitalists’ Priorities for Inclusive Workplace Strategies

**DOI:** 10.7759/cureus.89816

**Published:** 2025-08-11

**Authors:** Hirotaka Kato, Maria I Del Castillo De Calvo, Waliah Muhammad, Celia E Castellanos, Zabu Myint Aung, Mahmoud Amr, Chunling Niu, Clifford A Kaye, Joseph R Sweigart

**Affiliations:** 1 Division of Hospital Medicine, Department of Internal Medicine, University of Kentucky College of Medicine, Lexington, USA; 2 Division of Hospital Medicine, Georgetown Community Hospital, Georgetown, USA; 3 Department of Graduate Studies, University of the Incarnate Word School of Education, San Antonio, USA

**Keywords:** belonging, hospitalist, inclusion, survey, unfolding

## Abstract

Introduction

We aimed to identify priority areas among strategies to foster an inclusive and engaging work environment and to examine how these strategies relate to one another through a needs assessment in a large hospital medicine group.

Methods

We conducted a secondary analysis of an anonymous survey administered in February 2023 at the University of Kentucky, a university hospital in the southern United States. A total of 85 respondents at the Division of Hospital Medicine ranked nine key strategies by urgency, including recruitment, retention, educational opportunities, belonging, psychological safety, inclusive workplace, inclusive policies, equity in care, and opportunities for collaboration. We used unfolding multidimensional scaling (UMDS) to visualize the relationships among respondents and strategies.

Results

Of the 85 complete responses, the respondents were primarily physicians (60 (70%)), White (51 (60%)), and women (43 (51%)). Retention (3.6±2.6) and belongingness (4.1±2.6) had the lowest (i.e., highest priority) mean ranks. The UMDS plot suggested one dimension spanning from diversity to inclusivity and the other from organizational to interpersonal continuum. Belonging and psychological safety clustered in the interpersonal-inclusivity domain, while recruitment, retention, and collaboration clustered in the interpersonal-diversity domain. Inclusive workplace, inclusive policies, and equity in care were aligned within the organizational-inclusivity domain. Education was an outlier, suggesting varied interpretations of its importance.

Conclusion

Belonging emerged as a high-priority strategy closely linked with psychological safety, suggesting its role in workforce inclusivity and engagement. Fostering belonging may support retention and promote a more inclusive culture in academic hospital medicine. Clarifying the definition and measurement of belonging can enhance its integration into institutional strategies.

## Introduction

The rapid growth of hospital medicine, especially in academic settings, has posed challenges to maintaining engagement within and amongst members of large hospitalist groups. Amid the physician burnout epidemic [1], fewer organic interactions among peers may lead to reduced peer support and increased sense of isolation [2]. This can result in significantly reduced job satisfaction and increased turnover. Factors such as longer hours and higher mental and physical demands may exacerbate burnout in the inpatient setting [3]. The consequences are severe. Increased turnover costs the nation an estimated $4.6 billion annually [4]. Physician engagement, which encompasses the domains of vigor, dedication, and absorption in work, is an antithesis of burnout [5]. An important driver of this engagement is belonging to a vibrant community, as relationships with colleagues have been correlated with career success and satisfaction [6]. In fact, collegial support and an inclusive team climate are crucial for fostering a sense of belonging among clinicians [7,8] and are associated with job satisfaction [9].

Neglecting hospitalists’ need for belonging, collegial support, and an inclusive climate can have negative downstream effects on patient care and medical education. However, creating an engaging and inclusive workplace culture can be challenging in hospital medicine due to factors such as large group sizes, a lack of senior mentors, and limited funding opportunities for extra-clinical areas of interest. At a bare minimum, we must explicitly discuss the challenges we face, setting belonging or related constructs as key elements of our mission, and sharing ideas to deepen our understanding of such constructs beyond our field [10]. Furthermore, addressing these issues requires a shift from individual-focused efforts to organizational and system-level interventions [11]. To develop effective strategies, it is crucial to first understand the specific needs and priorities of hospitalists themselves. Identifying high-yield opportunities for promoting a more inclusive and engaging workplace environment is a critical first step.

Our overarching goal is to aid explicit discussions about improving toward an engaging and inclusive work environment in hospital medicine. The purpose of our study is to assess 1) the priorities in improvement strategies and 2) the relationships among the strategies, as identified through a needs assessment conducted at a large academic hospital medicine group. To explore the complex relationships and perceptual relevance among improvement strategies, we employed an exploratory data analysis technique called unfolding multidimensional scaling (UMDS). 

## Materials and methods

Study design and setting

We conducted a secondary analysis of anonymous needs assessment survey data collected in February 2023 from the Division of Hospital Medicine (DHM) at the University of Kentucky, a large academic hospital medicine group in the southern United States. The DHM provides inpatient care across both a 569-bed quaternary care hospital and a 160-bed community hospital.

Study population and sample size

The DHM workforce primarily consists of physicians, advanced practice providers, and administrators. We included all 85 complete responses in the dataset for analysis. Because the division was expanding rapidly, a fixed roster for the February 2023 survey period was not retained. Scheduling records indicate that approximately 100-110 staff were available for the survey during the survey period, giving an estimated response rate of about 80%.

Survey measures

In 2022, a director was appointed to promote community advancement and engagement within DHM. Opportunities and improvement strategies were identified through a targeted literature review and further analyzed to align with local needs through two unstructured focus groups with division members, and unstructured interviews with leaders from the DHM, the hospital, and the College of Medicine. Nine key improvement strategies were identified and subsequently used as survey items. In February 2023, the DHM administered an anonymous needs assessment survey to identify high-yield opportunities for promoting an inclusive and engaging workplace culture.

In addition to demographic data (e.g., gender, race, Hispanic ethnicity, and years spent with DHM), the final survey asked respondents to rank the nine strategies by urgency, with 1 being the most urgent, and no ties permitted. The items included improving recruitment (referred to as Recruitment); improving retention (Retention); implementing diversity, equity, and inclusion education or curriculum for hospitalists (Educational Opportunities); improving the sense of belonging (Belonging); building psychological safety (Psychological Safety); working toward an inclusive workplace (Inclusive Workplace); working toward inclusive policies (Inclusive Policies); identifying and addressing gaps and biases in care (Equity in Care); and increasing improvement efforts in collaboration with units and other departments (Opportunities for Collaboration). The full survey instrument is available in Table 2 in the Appendix.

The draft instrument was reviewed by the division leadership team, a group of six hospitalists, to support the validity of the content and response process. Only minor wording adjustments were made. In February 2023, the DHM sent an anonymous survey link to all division staff members (excluding those who planned to leave at the end of June) via an online survey platform. Responses were collected for two weeks, with automated email reminders sent every 48 hours until survey completion (i.e., those who completed the survey stopped receiving this automated reminder).

Ethics statement

The university’s institutional review board determined this secondary analysis of existing de-identified survey data to be exempt from full review (Protocol #88988). As the data had already been collected and anonymous, the requirement for informed consent was waived.

Statistical analysis

Analyzing ranked responses offers advantages in assessing patterns of item rankings and people's preferences. We visually assessed the proximity between respondents and strategies, as well as between strategies, using UMDS, a type of dimension reduction approach. UMDS maps both the respondents and the ranked items on the same plot, yielding a visual representation of how individual respondents tend to prioritize the nine strategies. On the UMDS plot, we identified clusters of similar strategies and qualitatively interpreted the meaning of each dimension (x-axis and y-axis). The interpretations were finalized through a collaborative discussion among the research team. This helps elucidate how hospitalists collectively group and conceptualize areas for improvement, thereby informing potential targeted interventions. A significant improvement in stress value by permutation test suggested a reasonable goodness of fit (p < 0.01) [12]. The R package ‘smacof’ was used for the UMDS analysis.

## Results

Participant characteristics

The dataset consisted of 85 complete responses. The respondents were predominantly physicians (60 (70%)), White (51 (60%)), and women (43 (51%)). More than half of the respondents (48 (57%)) were new to the division within three years. Retention received the lowest mean (highest priority) rank at 3.64 ± 2.55, followed by belongingness at 4.06 ± 2.59 (see Table 1).

**Table 1:** Descriptive statistics: demographics and mean ranks of improvement priorities Abbreviations: APP: advanced practice provider, DHM: Division of Hospital Medicine, Std: standard deviation

Variable	f		(%)
Gender			
Man	39		(46%)
Woman	43		(51%)
Other	3		(3%)
Profession			
Physician	60		(70%)
APP	15		(18%)
Other	10		(12%)
Ethnicity			
Hispanic	2		(2%)
Race			
White	51		(60%)
Asian	23		(27%)
Black	1		(1%)
Other	10		(12%)
Years since joining DHM			
Three years or less	48		(57%)
Four to six years	16		(19%)
Seven years or more	20		(23%)
Prefer not to answer	1		(1%)
Mean rank of priorities		Mean	(Std)
Retention	3.64		(2.55)
Belonging	4.06		(2.59)
Collaboration	4.58		(2.68)
Inclusive policies	5.01		(2.27)
Inclusive workspace	5.02		(2.21)
Equity in care	5.15		(2.43)
Psychological safety	5.65		(2.27)
Recruitment	5.78		(2.68)
Educational opportunities	6.12		(2.56)

UMDS plot

**Figure 1:**
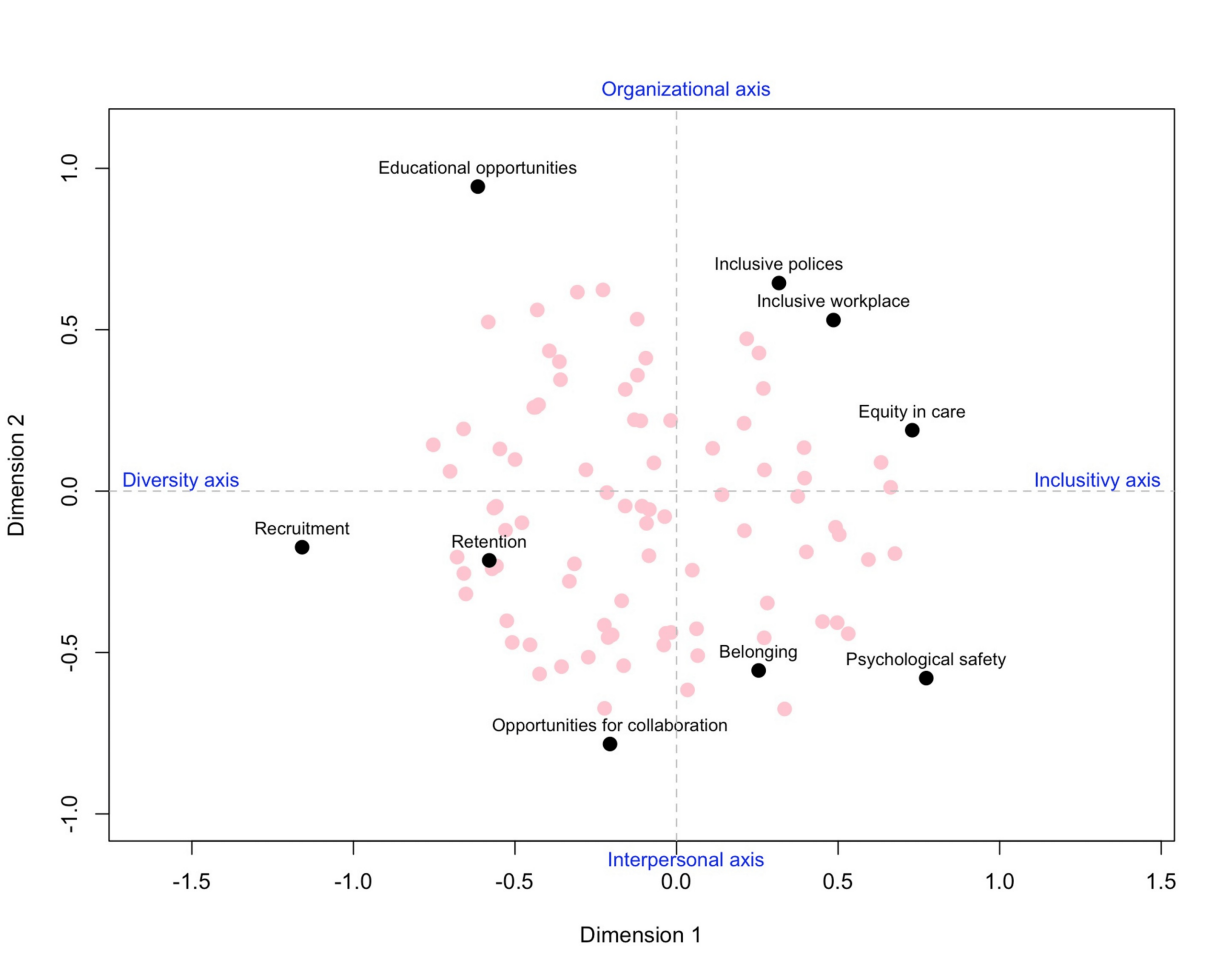
Unfolding multidimensional scaling: relationships among priorities in inclusive workplace strategies

We interpreted the key themes from the UMDS plot (Figure 1) as follows: the X-axis represents a spectrum from diversity to inclusivity, while the Y-axis captures an organizational to interpersonal orientation. Belonging and psychological safety are clustered in the interpersonal and inclusivity domain, while recruitment, retention, and collaboration are positioned in the interpersonal and diversity domain. Inclusive workplace, policies, and equity in care align within the organizational and inclusivity domain. Educational opportunities appeared as an outlier, indicating a lower preference. This may be attributed to measurement error, as some respondents interpreted the question as pertaining to receiving education, while others understood it as willingness to teach these topics. Overall, many respondents were situated somewhere between retention and belonging, indicating their relative prioritization.

## Discussion

In this division-wide needs assessment survey, 85 hospitalists ranked nine strategies for promoting an inclusive and engaging workplace; improving retention and strengthening the sense of belonging emerged as the most highly desired areas for action. These two elements exist symbiotically, with each strengthening the other [13]. A stronger sense of belonging contributes to greater retention, and better retention leads to a stronger sense of belonging among colleagues. This is supported by research, indicating that belonging is a crucial element for engagement, satisfaction, and reduced burnout [5,6,9] and linked to an intention to leave academic institutions [14]. While hospitalists have diverse interests and needs, these findings suggest that initiatives aimed at strengthening a sense of belonging could result in longer, more productive, and more fulfilling careers.

The emergence of belonging as a top priority is not surprising given that a sense of belonging is a universal human need that impacts wellbeing, achievement behavior, and mental health [15]. In academic medicine, belonging has been linked to a diverse workforce, inclusivity, and retention [16], making it an essential element of institutional strategies to improve workplace culture and engagement. It is not surprising that belonging can predict the quality of relationships within a team, self-fulfillment at work, and autonomy/decision latitude [17]. Lane-McKinely and Turner-Essel define belonging as having multiple domains and multiple levels in different contexts; therefore, having multiple domains of belonging is necessary, as a single point of belonging can be vulnerable to any change [10]. 

Despite a prevalent use of belonging in the context of retention, burnout, and engagement, we found a paucity of available data around belonging, particularly for its measurement and improvement strategies. Our unique findings offer a novel, data-driven foundation for supporting explicit discussions, measurement, and improvement efforts directed at belonging, which is vital to improving job satisfaction and rightfully fostering inclusive workplace environments in the context of hospital medicine. It is noteworthy, however, that although we captured the perceived importance of belonging through our single-item ranking, this does not reflect a validated instrument for measuring belonging.

While belonging is increasingly recognized as an essential element of well-being, it remains under-conceptualized in academic medicine. A consistent definition of belonging, emphasizing its multi-layered nature, influencing both interpersonal relationships and organizational culture, can help institutions better integrate belonging into broader efforts to support inclusive and equitable work environments. Future work could incorporate established scales or develop structured assessments to more accurately benchmark belonging across different institutions.

Implications and next steps in hospital medicine

Belonging is particularly important in hospital medicine, which often involves shift work, large teams, and rotating staff-factors that can hamper interpersonal continuity. Explicitly prioritizing belonging in local organizational efforts may foster an inclusive environment, thus mitigating burnout and turnover while promoting patient care quality. As our UMDS analysis suggests, belonging was closely intertwined with psychological safety, emphasizing calls for organizational approaches that address interpersonal needs and cultural norms simultaneously.

Efforts to achieve these goals are already underway at various levels. At the institutional level, emotional and financial support for initiatives focused on workforce engagement and inclusion is a critical determinant of success for such efforts. Designating local leaders to champion these initiatives ensures that these efforts receive adequate attention and resources [18]. Compass Groups, in which diverse groups of providers meet periodically for kinship and discussions, have been shown to help with retention and satisfaction [19]. Ensuring cultural and experiential diversity within such groups can further enhance their positive impacts by sharing their unique experiences and reducing unconscious bias among participants.

At the national level, professional societies also play an important role in recruiting and engaging a diverse workforce. The Society of Hospital Medicine (SHM) offers scholarships for medical students from underrepresented minority groups [20] and recognizes inclusive leadership through honors and awards [21]. Active participation in national societies also affords opportunities for individuals to expand their external networks and discover opportunities to sponsor and mentor junior faculty, such as through Special Interest Groups and the Visiting Professor Program [22]. Finally, authors and editors of medical literature can strengthen these efforts by refining definitions of belonging in academic literature and disseminating effective strategies to improve belonging in health professions.

While our findings provide valuable insights into belonging within a large academic hospital medicine group, they should be interpreted with certain limitations. First, the applicability of these results to other practice settings may be limited due to the single-center design with a relatively small sample and limited diverse backgrounds. In addition, inconsistent definitions of belonging in the literature present challenges to the generalizability of this and similar studies. Lastly, the results presented herein are solely based on a needs assessment and should not be interpreted as validations or evaluations of any specific improvement strategy. Addressing these limitations in future research will help build a stronger foundation for understanding and promoting belonging across diverse settings.

## Conclusions

Much work remains to be done to find effective strategies that foster inclusive and engaging work environments. It is useful for those involved to recognize that a sense of belonging plays a crucial role in these efforts. Moreover, further research and discussions are necessary to more precisely define and measure belonging in hospital medicine, as it directly affects patient care and healthcare systems. We hope that candid and transparent engagement with these topics will inspire broader participation across diverse backgrounds and practice settings, as cultural humility is a crucial first step toward creating an inclusive work alliance.

## Appendices

**Table 2:** Needs Assessment Survey Abbreviations: DHM: Division of Hospital Medicine, APP: advanced practice provider

Introduction		
We would like to understand your priorities among the following nine improvement strategies aimed at promoting inclusive and engaging workplace culture. Please reflect on your experiences with our division and think about which items are more or less urgent than others.		
Item (Strategy)		Example
Improve recruitment		(e.g., diversifying workforce, recruiting underrepresented groups, showing our values on DHM website, outreach efforts to recruit underrepresented groups)
Improve retention		(e.g., equitable opportunities for career advancement, more pipelines to professional networks and mentors within and outside DHM)
Implement diversity, equity, and inclusion education/curriculum for hospitalists		(e.g., having guest speakers, a series of seminars/workshops, learning modules, utilizing educational resources outside DHM)
Improve the sense of belonging		(e.g., more opportunities for personal and professional connections among us; celebrating professionally & culturally important days; building partnership between physician and APP hospitalists, more opportunities for APPs)
Build psychological safety		(e.g., creating a platform where people can safely share experiences and improvement ideas, such as biases & inequity toward staff and/or patients)
Work toward inclusive workspace		(e.g., lactation workroom, Halal/Kosher food)
Work toward inclusive policies		(e.g., transparent shift distribution & teaching assignment, reduce biases in patient care policies like care contract/administrative discharges)
Identify and address gaps and biases in care		(e.g., learning from individual staff & patient experiences and/or inpatient data)
Increase improvement efforts in collaboration with units/other departments		(e.g., launch various campaigns in medical units like words matter, MyChart access by vulnerable groups)
Question		
*Rank (prioritize) the following items from 1 to 9, 1 being the top priority in your opinion.		
( )	Improve recruitment	
( )	Improve retention	
( )	Implement diversity, equity, and inclusion education/curriculum for hospitalists	
( )	Improve the sense of belonging	
( )	Build psychological safety	
( )	Work toward inclusive workspace	
( )	Work toward inclusive policies	
( )	Identify and address gaps and biases in care	
( )	Increase improvement efforts in collaboration with units/other departments	
*Note: Since choice randomization is enabled, the initial order of items is randomized for each respondent.		
How would you describe yourself?		
( )	Men	
( )	Women	
( )	Non-binary	
( )	[Prefer to self-describe]	
( )	Prefer not to answer	
Are you of Hispanic, Latino, or Spanish origin?		
( )	Yes	
( )	No	
( )	Prefer not to answer	
How would you describe yourself?		
( )	Black or African-American	
( )	American Indian or Alaskan Native	
( )	Asian	
( )	Native Hawaiian or Other Pacific Islander	
( )	White	
( )	[Prefer to self-describe]	
( )	Prefer not to answer	
Please select which best applies to you:		
( )	Administrative Professional	
( )	Advanced Practice Provider (including APP resident)	
( )	Other (e.g., Nurse Navigator)	
( )	Physician	
( )	Prefer not to answer	
How many years have you been with the DHM?		
( )	1-3 years	
( )	4-6 years	
( )	7-9 years	
( )	10 years or longer	
( )	Prefer not to answer	
